# The Ergonomic Comparison of Endoscopist's Hand/Arm Movement and Relevant Muscle Load Between the Diagnostic and Therapeutic Upper Gastrointestinal Endoscopy

**DOI:** 10.1111/den.70078

**Published:** 2025-12-29

**Authors:** Shinnosuke Nagano, Kota Momose, Yuji Ishii, Shuhei Yamaguchi, Motoki Sasaki, Sho Komukai, Kotaro Yamashita, Takuro Saito, Koji Tanaka, Tomoki Makino, Tsuyoshi Takahashi, Yukinori Kurokawa, Hidetoshi Eguchi, Yuichiro Doki, Kiyokazu Nakajima

**Affiliations:** ^1^ Department of Next Generation Endoscopic Intervention (Project ENGINE) The University of Osaka Graduate School of Medicine Osaka Japan; ^2^ Department of Gastroenterological Surgery The University of Osaka Graduate School of Medicine Osaka Japan; ^3^ Castem Co., Ltd Hiroshima Japan; ^4^ Division of Research and Development for Minimally Invasive Treatment, Cancer Center Keio University School of Medicine Tokyo Japan; ^5^ Department of Health Data Science Tokyo Medical University Tokyo Japan

**Keywords:** endoscopic submucosal dissection, ergonomics of endoscopy, esophagogastroduodenoscopy, motion capture analysis, muscle activation

## Abstract

**Objectives:**

There have been little quantitative studies of ergonomics in the upper gastrointestinal endoscopy. We aimed to identify the ergonomic characteristics of both diagnostic (esophagogastroduodenoscopy; EGD) and therapeutic (endoscopic submucosal dissection; ESD) endoscopy in terms of the movement range of the endoscopist's hand/arm holding the endoscope and muscle activity during procedures, in a preclinical setting.

**Methods:**

(1) Optical reflective markers attached to the standard flexible endoscope were traced using a multiple motion capture system during EGD and ESD. The data were processed to generate three‐dimensional XYZ coordinate data for each procedure. (2) Wireless electromyogram electrodes were attached to eight muscles in the left hand, forearm, shoulder, neck and back. Muscle activation during EGD and ESD was assessed as % maximal voluntary contraction (%MVC).

**Results:**

(1) The motion capture was performed during 13 EGDs and 12 ESDs. On all XYZ axes, the movement range of the endoscope was significantly smaller during ESD than EGD (X; *p* < 0.001, Y; *p* = 0.015, Z; *p* < 0.001). (2) The EMG was recorded during 15 EGDs and eight ESDs. The higher mean %MVC of the pronator teres muscle (52.1%) and the extensor carpi radialis muscle (39.3%) was observed during all procedures. The %MVC tended to be higher during ESD (34.1%) than EGD (28.9%) in an analysis including all muscles (*p* = 0.078).

**Conclusions:**

Our study is the first to show therapeutic endoscopy had the smaller movement range of the endoscope, but the larger muscle activity than diagnostic endoscopy. These data could deepen our ergonomic understanding of endoscopy and help to optimize endoscopic techniques and/or relevant working environment.

## Graphical abstract

Ergonomic comparison of hand/arm movements and muscle load in diagnostic vs. therapeutic upper GI endoscopy.

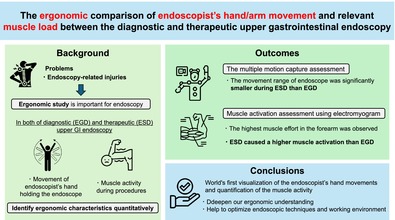

## 1 Introduction

Ergonomics is the study of the physical and cognitive demands of a task in relation to an individual's capacity [1]. Ergonomic studies in technical medical procedures are essential to optimize the work environment and reduce workload and musculoskeletal disorders [1]. For example, in laparoscopic and robotic surgery, ergonomic studies have evaluated forceps movements using motion capture [2, 3, 4, 5] and muscle activity using electromyogram (EMG) [6, 7]. These findings have informed improvements in surgical equipment and operating room design. However, ergonomic research in flexible endoscopy, especially upper gastrointestinal (GI) endoscopy, remains limited.

Endoscopists spend over 40% of their working hours engaged in endoscopic work [8, 9], and survey‐based studies reported a certain prevalence of endoscopy‐related injuries (ERIs) in gastroenterologists [10]. Long‐term ERIs may disrupt endoscopists' daily lives, limit practice due to pain, and lead to loss of skilled workforce [8, 11]. The recent expansion of cancer screening and endoscopic treatments will increase both diagnostic and therapeutic procedures volume and endoscopists' workload, requiring urgent countermeasures [11, 12, 13].

Therapeutic endoscopy has become increasingly advanced and time‐consuming recently, placing a greater mental and physical burden on endoscopists than diagnostic procedures [8, 14, 15, 16]. Although diagnostic and therapeutic endoscopies have different characteristics in terms of their technical aspects, little has been studied about the difference of ergonomics between diagnostic and therapeutic endoscopy. We considered that it would be significant to clarify the ergonomic characteristics of both diagnostic and therapeutic endoscopy for a deeper understanding of each technique and optimizing the work environment and equipment.

The aims of this study were to obtain quantitative ergonomic data during the upper GI endoscopy, and to clarify the ergonomic characteristics of both diagnostic and therapeutic endoscopy focusing on the movement of the endoscopist's left hand holding the control section of the endoscope and the muscle activity in the left upper limbs, neck and back during the procedures.

## 2 Methods

### 2.1 Participants

We recruited clinical endoscopists to collect data. We classified participants into two groups: an “expert”, who experienced performing endoscopic submucosal dissection (ESD) in over 100 cases and is a board‐certified physician of the Japan Gastroenterological Endoscopy Society, and a “novice”, who did not meet this criteria and had limited experience performing upper endoscopy in clinical practice.

### 2.2 Endoscopic Procedures

All endoscopic procedures were performed on female live swine models weighing approximately 35 kg under general anesthesia, using a standard flexible endoscope (GIF‐Q260J; Olympus, Tokyo, Japan). We adopted esophagogastroduodenoscopy (EGD) as a diagnostic upper gastrointestinal endoscopy and ESD for the posterior wall of the esophagus or gastric body as a therapeutic endoscopy. For data acquisition, each endoscopic procedure was performed only once per endoscopist. During EGD, participants observed the esophagus and stomach (gastric body and antrum), taking photographs according to each endoscopist's usual clinical workflow. After reaching the pyloric ring, the cardia was examined by retroflexing the endoscope. Finally, the endoscope was withdrawn. An independent observer assessed whether each endoscopic site was reached during the procedure. For ESD, the procedure consisted of three steps: (1) mucosal marking, (2) submucosal injection, and (3) mucosal incision and submucosal dissection. First, mucosal marking was performed using a 1.5‐mm electrocautery device (DualKnife J, KD‐655Q; Olympus, Tokyo, Japan) to create a virtual lesion of approximately 20 mm. Next, normal saline mixed with a small amount of indigo carmine was injected into the submucosal layer using endoscopic puncture needles (01963; TOP Corp., Tokyo, Japan). Finally, mucosal incision and submucosal dissection were carried out using the DualKnife J, and completion was defined as the retrieval of the specimen outside the body and macroscopic confirmation of the specimen.

The endoscopy room was arranged according to the American Society for Gastrointestinal Endoscopy (ASGE) ergonomic guidelines [10, 17]. The sub‐monitor was positioned directly in front of the endoscopist, 15°–25° below eye level at a viewing distance of about 80 cm. The patient bed height was adjustable from 85 cm to 120 cm.

### 2.3 The Multiple Motion Capture Assessment

**FIGURE 1:**
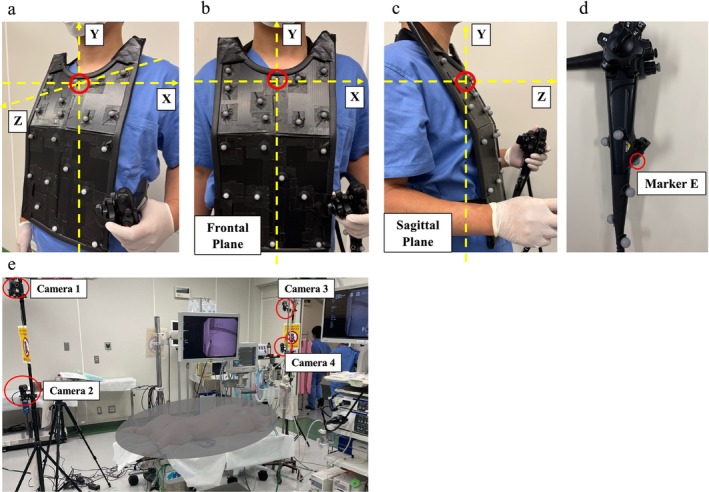
The setting of the multiple motion capture assessment. (a) The setting of the reflective markers. The origin was set at the sternal manubrium (red circle). The body's right‐to‐left axis was defined as the X‐axis, the cranial‐to‐caudal axis as the Y‐axis, and the anterior‐to‐posterior axis as the Z‐axis. The positive direction of the X‐axis corresponds to the operator's left side, the Y‐axis to the operator's head side, and the Z‐axis to the operator's front. (b) The X–Y plane was defined as the frontal plane. (c) The Y–Z plane was defined as the sagittal plane. (d) The marker attached 84 mm above the lower end of the endoscope body was defined as the representative point of the endoscope (Marker E). (e) The setting of the four infrared optical motion capture cameras.

We used an optical motion capture system (OptiTrack Flex13; NaturalPoint, OR) for our data collection. This system uses multiple infrared cameras to track the reflective markers attached to the whole body and objects, and automatically creates a skeletal model. Then, the movement of the object relative to the body can be expressed as 3D coordinates. In this study, we attached six reflective markers to the endoscope body, and the endoscopist wore the jacket with 20 markers (Figure 1a–d). A total of four infrared optical motion capture cameras at 100 Hz were used for motion tracking and recording the coordinates of each reflective marker (Figure 1e).

### 2.4 The Movement Range of the Endoscope

The marker attached 84 mm above the lower end of the endoscope was defined as the representative point of the endoscope (Marker E, Figure 1d). The movement of this marker was analyzed as the movement of the endoscopist's left hand holding the endoscope. The coordinate data of Marker E was reconstructed and converted to 10 Hz using the data from all 26 reflective markers. The data were repositioned in the 3D coordinates with the sternal manubrium as the origin. The body's right‐to‐left axis was set as the X‐axis, the cranial‐to‐caudal axis as the Y‐axis, and the anterior‐to‐posterior axis as the Z‐axis (Figure 1a). We plotted the coordinate data on two planes: the X–Y plane as the frontal plane and the Y–Z plane as the sagittal plane to visualize endoscope movement (Figure 1b,c). EGD and ESD were performed using this motion capture system, and mean (SD) coordinates from the origin for each procedure were calculated along each axis.

### 2.5 The Angulation Range of the Endoscope

With regard to the angle of the endoscope body, the slope of the straight line connecting the Marker E and the marker at the bottom end of the endoscope was calculated for all 3D coordinate plots. The slope was translated into the angle relative to the X‐axis in the frontal plane and the angle relative to the Z‐axis in the sagittal plane. The mean (SD) of the angle was calculated during EGD and ESD.

### 2.6 Muscle Activation and Effort

Objective muscle activation was assessed using wireless surface EMG (BioLog DL‐5500, DL‐510A; S&ME, Tokyo, Japan). We recorded eight muscle activations: the left biceps brachii muscle, trapezius muscle, extensor carpi radialis muscle, flexor carpi ulnaris muscle, pronator teres muscle, thenar muscle, back neck muscle, and erector spinae muscle. First, eight EMG sensors were attached to the endoscopists, and the resting EMG was recorded [18, 19]. Detailed EMG sensor placement sites are provided in Table S1. Before endoscopic procedures, the maximal voluntary contraction (MVC) of each muscle was recorded for 5 s by maximally resisting appropriate joint motions, which were kept at a “5/5 (normal)” MMT level and best isolated each muscle [20]. To standardize measurements across participants, all MVC assessments were performed by the same examiner; participants were positioned consistently. Raw EMG signals were collected at a sampling rate of 1000 Hz and processed using a band‐pass filter of 5–200 Hz with a −20 dB/decade roll‐off to remove movement artifact and high‐frequency noise. Next, root‐mean‐square (RMS) values were computed as the square root of the mean of squared EMG amplitudes within the analysis window. All RMS amplitudes were then expressed as a percentage of MVC (%MVC = (RMS/MVC) × 100) [6]. A higher %MVC indicates higher muscle activation [7].

### 2.7 Statistical Analysis

Statistical analyses were conducted using JMP software (version 17.0.0; SAS Institute, Cary, NC). Categorical and continuous variables between the EGD and ESD groups were compared using the chi‐square test and Mann–Whitney *U* test, respectively. Motion capture data (coordinates and angles) were expressed as mean ± 2SD, and differences in the range of scope movement and angulation were analyzed using the Wilcoxon rank sum test on 2SD values for each surgeon. %MVC was expressed as mean ± SE, and comparisons between EGD and ESD were performed by linear regression with surgeon and muscle as covariates. A *p*‐value < 0.05 was considered statistically significant.

## 3 Results

### 3.1 The Movement Range of the Endoscope

**FIGURE 2:**
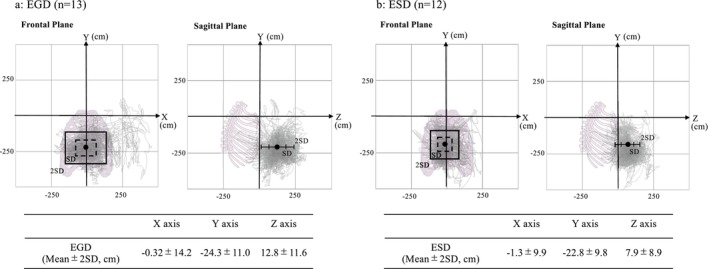
The movement range of the endoscopist's left hand holding the endoscope during EGD (a) and ESD (b). For each EGD (a) and ESD (b) procedure, the trajectories of Marker E, attached 84 mm above the lower end of the endoscope body, coordinates for all participants were plotted in the figure as light gray dots. These dots were projected onto the XY plane (Frontal plane) and YZ plane (Sagittal plane), as shown in the figure. The pink human skeleton indicates the operator's body orientation in each respective plane. The large black dots showed the mean coordinates of all the plots. The range of standard deviation (SD) and 2SD of the plotted coordinates was shown in the figure. EGD, esophagogastroduodenoscopy; ESD, endoscopic submucosal dissection.

**FIGURE 3:**
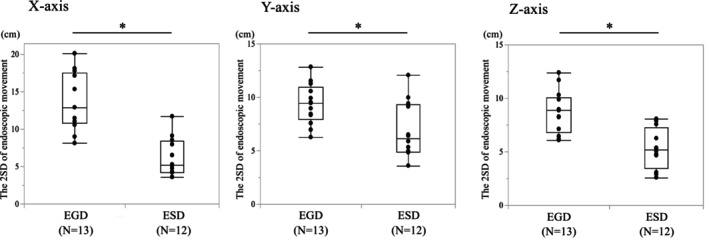
Comparison of the 2SD for the endoscopic movement between EGD and ESD. The movement range of endoscopist's left hand holding the endoscope were compared between EGD and ESD for each three axis. Wilcoxon rank sum test was used for 2SD for the endoscopic movement of each surgeon. *p < 0.05. EGD, esophagogastroduodenoscopy; ESD, endoscopic submucosal dissection.

A total of 17 endoscopists participated in the motion capture assessment, of whom 8 performed both EGD and ESD, providing motion capture data for 13 EGD and 12 ESD procedures (Table S2a). For these procedures, we obtained a total of 142,996 points of coordinate data: 39,687 points for EGD, 103,309 points for ESD. All coordinate data from each EGD and ESD, along with mean X, Y, and Z values during the procedures, were shown in Figure 2a,b. Comparing the 2SD of the movement range of the endoscope for each participant between EGD and ESD, the range of movement was significantly smaller during ESD than EGD on all three axes (X; *p* < 0.001, Y; *p* = 0.015, Z; *p* < 0.001, Figure 3).

### 3.2 The Angulation Range of the Endoscope

**FIGURE 4:**
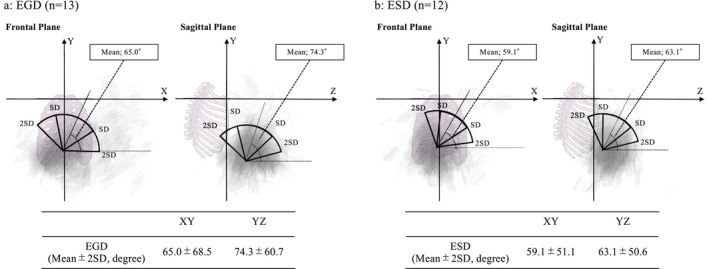
The angulation range of the endoscope body during EGD (a) and ESD (b). For each EGD (a) and ESD (b) procedure, all the slopes of the straight lines connecting Marker E and the marker at the bottom end of the endoscope body are shown in the figure as thin gray lines. These lines were projected onto the XY plane (frontal plane) and YZ plane (sagittal plane), as illustrated. The pink human skeleton indicates the operator's body orientation in each respective plane. All the corrected slope was expressed as the angle relative to the X‐axis in the frontal plane and the angle relative to the Z‐axis in the sagittal plane. The mean ± standard deviation (SD) of the angle was calculated during EGD and ESD. The range of SD and 2SD of the endoscopic angulation was shown in the figure. EGD, esophagogastroduodenoscopy; ESD, endoscopic submucosal dissection.

All the slope of the straight line connecting the marker E and the marker at the bottom end of the endoscope was obtained from 13 EGD and 12 ESD procedures, and the mean values were shown in Figure 4a,b. Comparing the 2SD of endoscope angulation between EGD and ESD, the range of the angulation relative to the Z‐axis in the sagittal plane showed significantly smaller in the ESD (*p* = 0.007, Figure S1a). On the other hand, there was no statistical difference in the angulation relative to the X‐axis in the frontal plane between the two groups (*p* = 0.12, Figure S1b).

### 3.3 Muscle Activation and Effort

**FIGURE 5:**
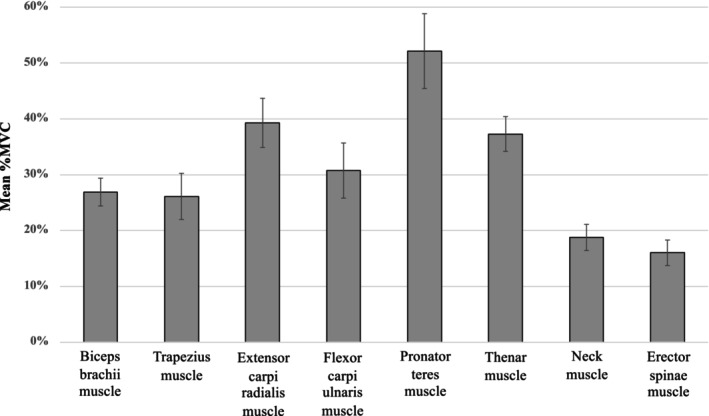
The mean %MVC of the eight muscles during the total of 23 endoscopic procedures. %MVC was the root‐mean‐square electromyography value normalized to the MVC. %MVC was expressed as mean ± standard error (SE). MVC, maximal voluntary contraction.

A total of 15 endoscopists participated in the assessment for muscle effort during endoscopy, of whom 8 performed both EGD and ESD, providing data for 15 EGD and 8 ESD procedures (Table S2b). The mean %MVC of eight muscles during a total of 23 procedures (EGD and ESD) was shown in Figure 5. Among these muscles, the pronator teres showed the highest mean %MVC at 52.1% ± 6.7%, followed by the extensor carpi radialis at 39.3% ± 4.4%, and then the thenar muscle at 37.3% ± 3.1%. The mean % MVC of the neck muscle (18.8% ± 2.3%) and erector spinae muscle (16.0% ± 2.3%) was lower than those of the other muscles.

**FIGURE 6:**
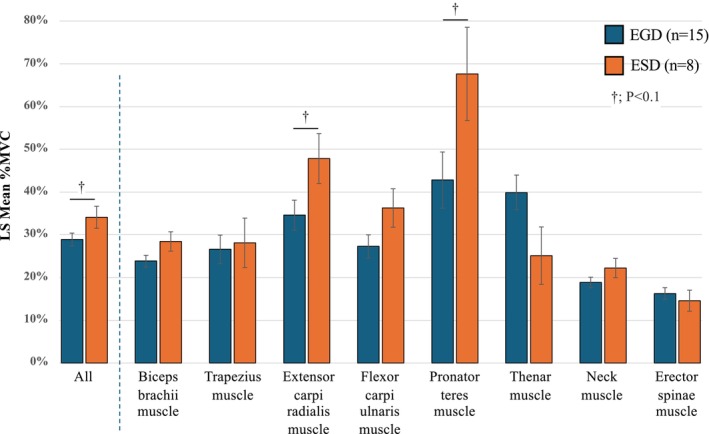
Comparison of the Least Square (LS) mean %MVC between EGD and ESD. Least Square (LS) mean %MVC of the eight muscles was compared between EGD and ESD. %MVC was the root‐mean‐square electromyography value normalized to the MVC. %MVC was expressed as mean ± standard error (SE). †*p* < 0.10. EGD, esophagogastroduodenoscopy; ESD, endoscopic submucosal dissection; MVC, maximal voluntary contraction.

The results of comparing the Least Square (LS) mean %MVC between EGD and ESD were shown in Figure 6. Evaluation of LS mean %MVC for all eight muscles between EGD and ESD revealed that muscle activation during ESD tended to be higher than that during EGD (28.9% ± 2.6% vs. 34.1% ± 1.5%, *p* = 0.078). Of each of the eight muscles, LS mean %MVC of the extensor carpi radialis muscle (34.6% ± 3.5% vs. 47.8% ± 5.8%, *p* = 0.093) and pronator teres muscle (42.8% ± 6.6% vs. 67.6% ± 10.9%, *p* = 0.092) particularly tended to be higher in ESD compared to those in EGD. The results of muscle activation during three steps of ESD were shown in Figure S2; the mean %MVC was highest during the incision and dissection phase across all eight muscles.

## 4 Discussion

To our understanding, most previous flexible endoscopy research on ergonomics has focused only on ERIs [8, 11, 21, 22, 23, 24, 25]. It is certainly important to study the prevalence of musculoskeletal disorders and ERIs' risk factors. However, there has been no research focusing on the endoscopist's hand/arm movement, which is one aspect of ergonomics. This is the first report to quantitatively demonstrate that the endoscope's movement range and angulation are markedly smaller during ESD than during EGD. Regarding movement patterns, a comparison of the mean coordinates on each axis showed that the left hand during ESD was positioned closer to the body than during EGD. Diagnostic endoscopy requires wide scope movement to observe multiple organs, whereas ESD involves minimal movement once the lesion is targeted and depends mainly on dialing, fine torque, and forceps control. Thus, ESD could be a more static procedure than EGD. Although endoscopists may have intuitively recognized this, our visual and quantitative data provide clearer insight into endoscopic technique and ergonomics.

While the risks of ERIs during colonoscopy have been studied using EMG [26, 27], this is the first study to quantify muscle activity during upper GI endoscopy. In our study, the higher %MVC of the extensor carpi radialis and pronator teres muscles was observed in upper GI endoscopy. These results were similar to recent questionnaire‐based studies showing a high prevalence of upper limb ERIs [21, 22, 23]. We also showed that neck and lower back muscle loads were lower than those in others. However, previous reports have shown a high prevalence of neck and back pain among endoscopists [8, 24, 25, 28]. This discrepancy may be due to our use of ergonomic settings based on guidelines [10, 17]. Furthermore, our study showed that three forearm muscles and the thenar muscle showed an over 30% MVC, which is defined as the threshold limit value (TLV) of the American Conference of Governmental Industrial Hygienists (ACGIH) in endoscopy [27, 29]. The ACGIH TLV is a model for estimating musculoskeletal risk in upper limbs during work; tasks exceeding TLV are high risk and require redesign [27]. Our data suggest that it is necessary to optimize the working environment and devices for both diagnostic and therapeutic endoscopies in upper GI endoscopy as with previous reports on colonoscopy [27].

Considering both the motion capture and EMG data, ESD involved a smaller range of endoscopic movement but, paradoxically, resulted in higher muscle activity than EGD. ESD is a highly technical procedure that involves various techniques, while maintaining an appropriate endoscopic view; the complexity of these techniques would have led to a larger muscle load for the endoscopists. Furthermore, the incision and dissection step showed the highest mean %MVC across all eight muscles, suggesting that it demands greater technical skill than mucosal marking or submucosal injection. As several studies have reported, fatigue during endoscopic procedures can reduce procedure quality [30, 31], posing a risk to patient outcomes. Our findings emphasize the need to address ergonomics for endoscopists performing more therapeutic procedures, by optimizing workstations, modifying procedures, redesigning the endoscope handle, and developing ergonomic devices or training programs [27, 32]. While ASGE guidelines and other ergonomic studies already recommend practical strategies such as proper monitor positioning, posture, breaks, and ergonomic education [10, 17, 33], few devices have been specifically designed with ergonomics in mind. We are currently developing an endoscope‐holding assist device for therapeutic endoscopy based on our motion capture data to reduce muscle load (Figure S3), and in the future, robotic‐assisted flexible endoscopes may further decrease endoscopist workload [34, 35, 36].

Comparing movement and muscle load between experts and novices is important. Our study suggested that experts had a smaller X‐axis movement range during diagnostic endoscopy, indicating more efficient scope control (Data not shown). In laparoscopic surgery, motion capture has been used to analyze expert movements and develop training systems [2, 3, 4, 5]. In flexible endoscopy, techniques vary by endoscopist, and standardization is lacking. With the rise of advanced procedures such as ESD [8, 14, 15, 16], analyzing expert movements and muscle activity could inform ergonomic educational tools and serve as indicators of skill proficiency for trainees. Further research is needed to compare expert and novice ergonomics for clinical use.

There are several limitations in this study. First, endoscopic procedures were performed in swine models and each procedure was performed only once. Second, although ESD was selected as the therapeutic procedure in this study, other upper GI endoscopic techniques were not evaluated. Third, muscle activity was described as tending to be higher during ESD than EGD when *p* < 0.1, considering the small sample size and the potential for stronger associations in larger studies. Fourth, regarding the sample size, the number of procedures in this study was determined by practical constraints rather than a formal a priori calculation, as the available participants and procedures were limited. Lastly, the difference in EGD and ESD duration may affect %MVC evaluation. While median frequency analysis is required to account for time‐dependent muscle fatigue [6, 7, 37], the present study involved single, relatively short procedures, so this analysis was not feasible. Further studies are needed to assess muscle fatigue during longer or repeated endoscopic procedures.

In conclusion, we conducted the world's first visualization of the endoscopist's hand/arm movements and quantification of the muscle activity during both the upper GI diagnostic and therapeutic endoscopies. Our data are expected to lead to a deeper understanding of the ergonomics of flexible endoscopes and to be applied to the development of new ergonomic assisted devices to reduce the endoscopist's burden.

## Author Contributions

Conception and design; S.N., K.M., Y.I., S.Y., M.S., and K.N. Analysis and interpretation of the data; S.N., K.M., Y.I., S.Y., S.K., and K.N. Drafting of the article; S.N., K.M., and K.N. Critical revision of the article for important intellectual content; K.Y., T.S., K.T., T.M., T.T., and Y.K. Final approval of the article; H.E., Y.D., and K.N.

## Funding

This work was supported by the Ministry of Economy, Trade and Industry (METI), Japan, through the R&D Support Program for Growth‐oriented Technology SMEs (Grant Number JPJ005698).

## Ethics Statement

All procedures in this study were performed in accordance with the ethical standards of the responsible committee on institutional animal experimentation.

## Consent

The authors have nothing to report.

## Conflicts of Interest

The authors declare no conflicts of interest.

## Supporting information


**Table S1:** Electromyogram sensor placement sites.
**Table S2:** (a) The characteristics of participants in the multiple motion capture assessment. (b) The characteristics of participants in the analysis of the muscle activation and effort.
**Figure S1:** Comparison of the 2SD for the angulation of the endoscope body between EGD and ESD.
**Figure S2:** The results of the muscle activation analysis during three steps of ESD procedure.
**Figure S3:** Endoscope‐holding assist device developing currently based on our motion capture data.
