# Supplementary material for: The Ergonomic Comparison of Endoscopist's Hand/Arm Movement and Relevant Muscle Load Between the Diagnostic and Therapeutic Upper Gastrointestinal Endoscopy
Source: Dig Endosc. 2025 Dec 29;38(1):e70078. doi: 10.1111/den.70078 (PMC12746059; doi:10.1111/den.70078)

**Table S1. electromyogram sensor placement sites**

| **Muscle** | **Electromyogram sensor placement sites** |
| --- | --- |
| Biceps brachii muscle | Over the central belly of the biceps brachii muscle. |
| Trapezius muscle | 2 cm inferolateral to the midpoint between the acromion and the seven cervical vertebra (C7) |
| Extensor carpi radialis muscle | The proximal third of the line between the cubital fossa and the radial styloid process |
| Flexor carpi ulnaris muscle | Midpoint of the line from the medial epicondyle to the ulnar styloid process |
| Pronator teres muscle | 2 fingerbreadths distal to the midpoint of the line connecting the medial epicondyle of the humerus and the cubital fossa. |
| Thenar muscle  (Abductor pollicis brevis) | Over the muscle belly at the thenar eminence, near the base of the proximal phalanx of the thumb. |
| Back neck muscle  (Splenius capitis muscle) | 2 cm lateral to the spinous process of the second cervical vertebra (C2) |
| Erector spinae muscle | 3 cm lateral to the left of the spinous process of the third lumbar vertebra (L3) |

EMG, electromyogram

**Table S2.**

**a: The characteristics of participants in the multiple motion capture assessment**

|  |  | EGD  (n = 13) | ESD  (n = 12) | *P* |
| --- | --- | --- | --- | --- |
| Age (years) | Median (range) | 35 (31–55) | 34.5 (31–49) | 0.65 |
| Sex | Male / Female | 13 / 0 | 12 / 1 | 0.36 |
| Experience | Expert / Novice | 5 / 8 | 7 / 5 | 0.32 |
| ESD Location | Esophagus / Stomach | - | 2 / 10 | - |
| Time of the procedure (minutes) | Median (range) | 4.3 (2.5-18.7) | 16.0 (6.4-20.0) | <0.001 |

**b: The characteristics of participants in the analysis of the muscle activation and effort**

|  |  | EGD  (n = 15) | ESD  (n = 8) | *P* |
| --- | --- | --- | --- | --- |
| Age (years) | Median (range) | 40 (31–55) | 38 (31–47) | 0.60 |
| Sex | Male / Female | 13 / 2 | 7 / 1 | 0.96 |
| Height (cm) | Median (range) | 174 (157.5–185) | 173.5 (158–185) | 0.75 |
| Weight (kg) | Median (range) | 65 (45–80) | 64.5 (45–78) | 0.87 |
| Experience | Expert / Novice | 6 / 9 | 6 / 2 | 0.11 |
| ESD Location | Esophagus / Stomach | - | 0 / 8 | - |
| Time of the procedure (minutes) | Median (range) | 3.4 (2.0-7.4) | 22.5 (13.0-32.0) | <0.001 |
| MVC of biceps brachii muscle (µV/sec) | Median (range) | 233.6 (138.7–1038.9) | 206.8 (124.4–500.8) | 0.26 |
| MVC of trapezius  muscle (µV/sec) | Median (range) | 59.6 (16.4–521.9) | 60.6 (16.4–127.9) | 0.58 |
| MVC of extensor carpi radialis muscle (µV/sec) | Median (range) | 205.5 (82.0–441.2) | 207.1 (41.3–263.3) | 0.38 |
| MVC of flexor carpi  ulnaris muscle (µV/sec) | Median (range) | 199.4 (53.4–489.9) | 180.8 (53.4–265.6) | 0.26 |
| MVC of pronator teres  muscle (µV/sec) | Median (range) | 71 (31.5–129.9) | 61.5 (17.6–111.6) | 0.35 |
| MVC of thenar  muscle (µV/sec) | Median (range) | 632.7 (186.1–1419.0) | 407.6 (36–1359.4) | 0.23 |
| MVC of back neck  muscle (µV/sec) | Median (range) | 54.7 (23.8–112.1) | 68.4 (26.3–126.6) | 0.21 |
| MVC of erector spinae muscle (µV/sec) | Median (range) | 87.7 (27.7–162.4) | 79.4 (27.7–218.9) | 0.82 |

MVC, maximal voluntary contraction

**Fig. S1**

**Comparison of the 2SD for the angulation of the endoscope body between EGD and ESD**

The angulation range of the endoscope body from the X-axis (a) and the Z-axis (b) were compared between EGD and ESD. Wilcoxon rank sum test was used for 2SD for the angulation of the endoscope body of each surgeon. *P < 0.05.

EGD, esophagogastroduodenoscopy; ESD, endoscopic submucosal dissection


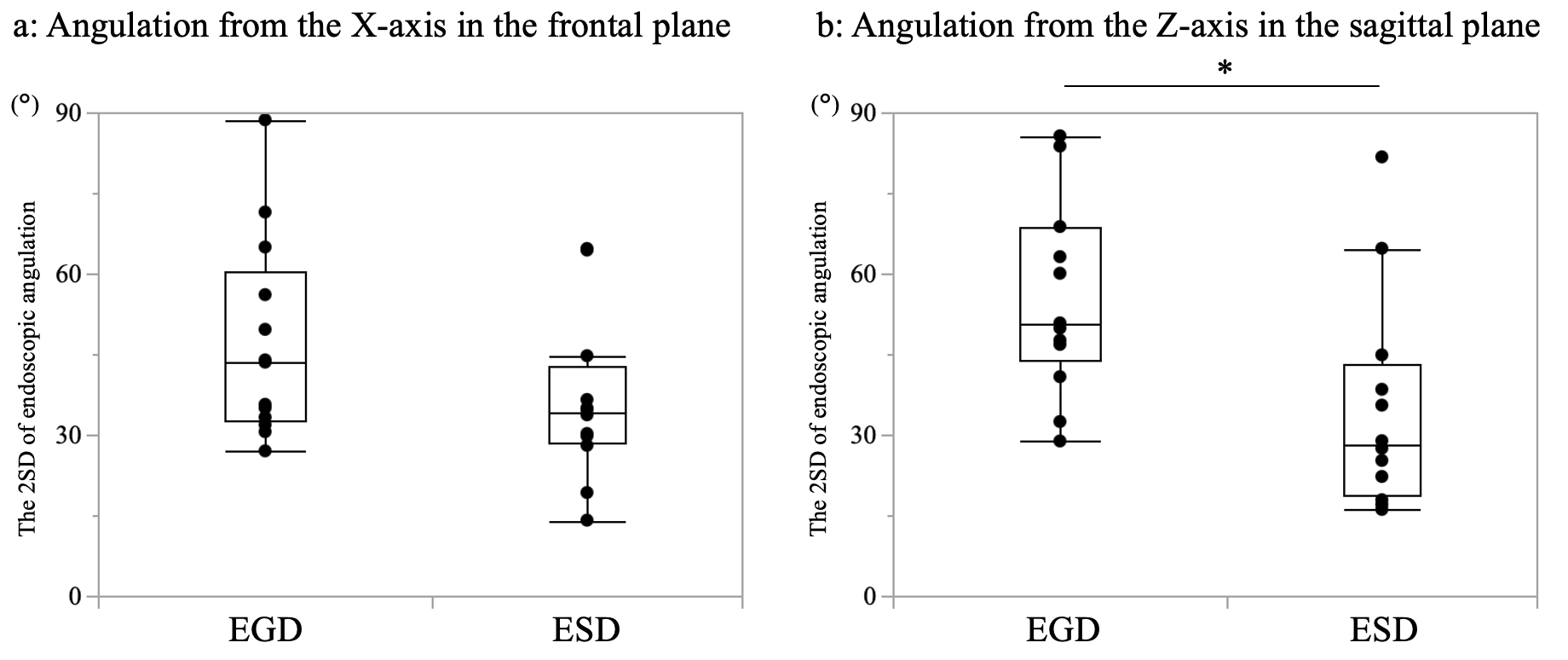


**Fig. S2**

**The results of the muscle activation analysis during three steps of ESD procedure**

The ESD procedure were divided to three steps: (1) the mucosal marking, (2) the submucosal injection, and (3) the mucosal incision and submucosal dissection. We analyzed muscle activity of the eight muscles during each step. The blue bar showed the mean %MVC of eight endoscopists for each step. The changes in muscle activity for each for each eight endoscopist were shown as a root-mean-square (RMS). %MVC was the RMS electromyography value normalized to the MVC.

ESD, endoscopic submucosal dissection; MVC, maximal voluntary contraction

**Fig. S3**

**Endoscope-holding assist device developing currently based on our motion capture data**


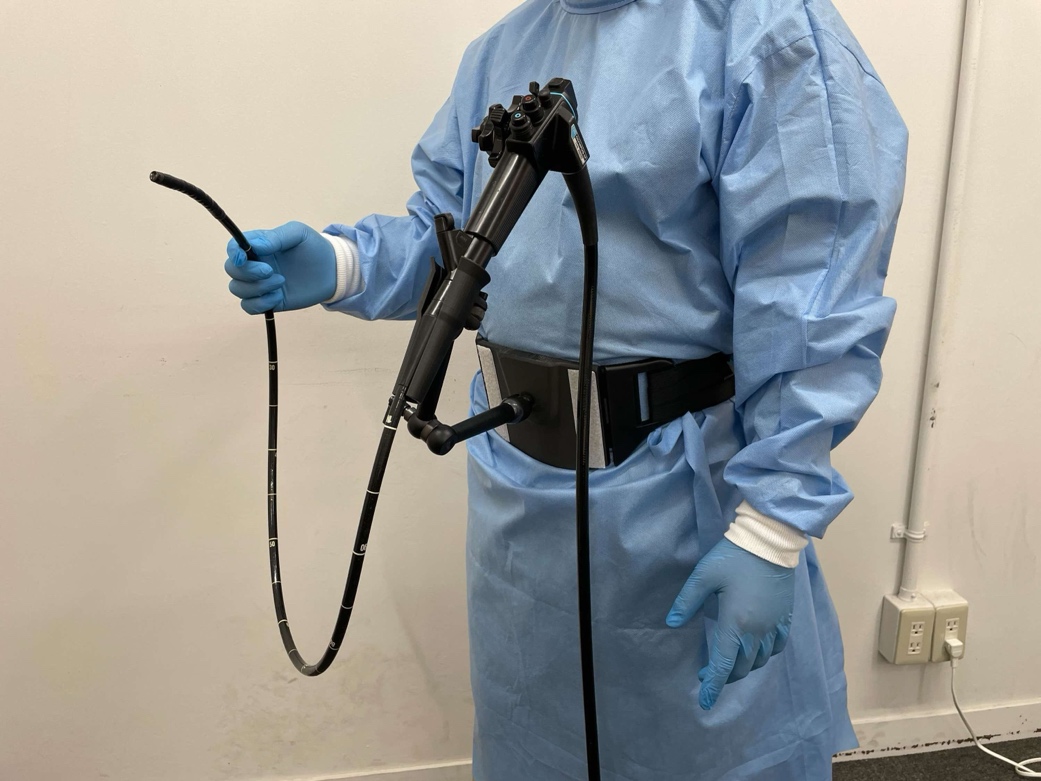

Supplement: Supplementary file 1 — Table S1: Electromyogram sensor placement sites. Table S2: (a) The characteristics of participants in the multiple motion capture assessment. (b) The characteristics of participants in the analysis of the muscle activation and effort. Figure S1: Comparison of the 2SD for the angulation of the endoscope body between EGD and ESD. Figure S2: The results of the muscle activation analysis during three steps of ESD procedure. Figure S3: Endoscope‐holding assist device developing currently based on our motion capture data. [file DEN-38-0-s001.docx]
